# Sagittal spinal curvatures of young adults in the context of their self-reported physical activity and somatic parameters

**DOI:** 10.1038/s41598-024-62929-9

**Published:** 2024-05-28

**Authors:** Małgorzata Grabara, Anna Witkowska

**Affiliations:** 1 Institute of Sport Science, Jerzy Kukuczka Academy of Physical Education, 72 Mikolowska Street, 40-065 Katowice, Poland; 2 Department of Health-Related Physical Activity and Tourism, Jerzy Kukuczka Academy of Physical Education, Katowice, Poland

**Keywords:** Thoracic kyphosis, Lumbar lordosis, Rippstein plurimeter, IPAQ, Health care, Public health

## Abstract

The objective of this study was to assess the thoracic kyphosis (ThKA) and lumbar lordosis (LLA) in healthy young adults and to investigate potential relationships between spinal curvatures, self-reported physical activity (PA), and somatic parameters. The study included 380 female students and 211 male students aged 20.7 ± 1.5 years. The ThKA and LLA were measured using a Plurimeter-V gravity inclinometer. The level of PA was estimated using the International Physical Activity Questionnaire. ThKA was lower in women compared to men, while LLA was higher in women than in men (p < 0.0001). Female students reported lower PA than male students (p < 0.001). Female students with ThKA within normal values reported a significantly higher amount of low-intensity PA compared to those with ThKA below or above the norm. A correlation was found between ThKA and body mass index (BMI), body adiposity index (BAI), WC, and fat percentage (rho < 0.2), whereas LLA showed correlations with BMI, BAI, waist circumference, and fat percentage (rho < 0.2). Among male students, a correlation was found between LLA and BMI as well as WC (rho < 0.2). Maintaining a healthy body composition may be instrumental in mitigating the risk of developing spinal curvature abnormalities.

## Introduction

Human posture depends on a variety of factors such as age, gender, somatic parameters, physical activity (PA), mental condition, occupation, condition of the muscular and nervous system, and genetic factors^1–7^. Body posture undergoes permanent changes, both during ontogenetic development and in the circadian rhythm. Normal posture is characterized by the symmetrical alignment of the pelvis, shoulders, and scapulae in the frontal and transverse planes. In the sagittal plane, the spine has four physiological curves, i.e. cervical and lumbar curves, which are concave (lordosis), and thoracic and sacral curves which are convex (kyphosis). These curvatures are geometric parameters that have an impact on the mechanical properties and load balance of the intervertebral tissues^6,8^. The normal shape of spinal curvatures is very important due to the load on passive spinal structures, pelvic alignment, and the prevention of pain. However, the assessment of the thoracic and lumbar curvatures is quite difficult, due to the lack of age- and gender-matched standards for both curvatures.

Abnormal thoracic kyphosis and/or lumbar lordosis may lead to the dynamic imbalance of the spine and impaired muscle tone^9^. This is potentially due to the disruption of normal biomechanical forces and alterations in the length-tension relationships of spinal muscles. Hyperkyphosis can be associated with the poor flexibility of chest muscles and hamstrings, low abdominal and paravertebral strength, and even the impairment of respiratory function, cervical pain, or subacromial pain syndrome^5,10–12^. These associations may be due to the altered spinal alignment causing compensatory changes in muscle length and tension, leading to muscle weakness and reduced flexibility. Hyperlordosis can be associated with the shortening of the psoas iliac, rectus femoris, and low abdominal and paravertebral strength. This could be due to the increased anterior pelvic tilt associated with hyperlordosis leading to adaptive shortening of hip flexor muscles such as the psoas iliac and rectus femoris. Hyperlordosis also may be linked to the high incidence of osteoarthritis in the hip and knee joints^5,10,13^.

A sedentary lifestyle and insufficient PA can cause the muscles supporting the spinal curvatures to weaken. This weakening can lead to postural abnormalities as the body struggles to maintain proper alignment without the necessary muscular support. An increase in the number of hours of prolonged sitting can contribute to a decrease in the lumbar lordosis angle^14^. This is due to the fact that prolonged sitting can lead to adaptive shortening of the hip flexor muscles, pulling the lumbar spine into a flattened position. On the other hand, undertaking PA on a regular basis can improve muscle strength and/or flexibility. This improvement can positively affect postural health by enhancing the body’s ability to maintain proper spinal alignment. Previous studies of adolescent and adult athlete populations have indicated that sports training can affect spinal curvatures due to specific positions and movements performed during training, with repetitive performance and specific movement patterns that can cause different adaptations in spinal curvatures^15–17^. This effect can vary depending on the sport and training intensity and can be more or less beneficial to posture. Other specific forms of PA or exercises programs such as physical yoga, Nordic walking, Pilates, and dancing can also influence posture^5,18–20^. These activities often focus on improving core strength and flexibility, both of which are crucial for maintaining proper spinal alignment.

Somatic parameters, referring to various physical characteristic of the human body, may be associated with the shape of sagittal spinal curvatures. Previous studies have shown that body weight, body mass, BMI, body adiposity index (BAI), waist circumference, total and central adiposity, or fat mass may affect thoracic kyphosis or/and lumbar lordosis angles^4,5,21–27^. However, these associations can be not very consistent and vary depending on the population studied, especially in terms of age and gender.

To the best of our knowledge, there has been a small number of studies specifically examining the associations between sagittal spinal curvatures, PA, and somatic parameters among young adults of similar age. Therefore, the objective of this study was to assess the physiological curvature of the thoracic kyphosis and lumbar lordosis in healthy young adults during the posture stabilization period and to investigate potential relationships between spinal curvatures, self-reported PA, and somatic parameters. We hypothesized that PA and somatic parameters may influence on spinal posture.

## Methods

### Participants

The required sample size for this investigation was determined using G*Power software, version 3.1.9.7, developed by Heinrich Heine University in Düsseldorf, Germany. The computation parameters included an anticipated effect size of 0.2, an alpha error probability of 0.05, and a test power of 0.95. Based on these specifications, the study required a total sample size of 495 participants. We invited 700 male and female students, randomly selected from all fields of study at the bachelor's level, considering a 30% a dropout rate. Ultimately, the study involved 380 women aged 20.7 ± 1.4 years and 211 men aged 20.7 ± 1.5 years, who were students of the Academy of Physical Education. The study inclusion criteria were as follows: (1) full-time undergraduate student status, (2) age between 18 and 30 years, (3) non-athlete, (4) no sports history, and (5) consent to participate in the study. The exclusion criteria included (1) previous injuries, illnesses, or diseases that restricted the ability to engage in PA, and (2) pregnancy.

### Methods and procedures

The study was approved by the Bioethics Committee of the Jerzy Kukuczka Academy of Physical Education in Katowice (No. 3/2012) and conformed to the standards established by the Declaration of Helsinki. All participants were informed about the type and aim of the study, and they provided informed consent before participating in the study.

Somatic parameters included body height (BH), body mass (BM), waist circumference (WC), and hip circumference (HC). The WC was measured as a minimum circumference between the iliac crest and the rib cage, and the HC was measured at the maximum width over the greater trochanters. Circumferences were measured over non-tight underwear or light-weight shorts using a tape measure while the participant stood upright, with feet together and arms hanging freely at the sides. BH was measured using an InBody BSM170 digital stadiometer (Biospace, Korea). BM and fat mass [%] were evaluated with an InBody570 analyzer (Biospace, Korea). The body mass index (BMI) was calculated based on BH and BM measurements using the formula: BMI = BM [kg]/BH [m^2^]. Standard BMI ranges were used to identify normal weight (18.6≤25), overweight (25≤30), and obesity (≥ 30)^28^. The body adiposity index (BAI) was calculated using the formula: BAI = HC [cm]/(BH [m])^^1.5^–18. BAI cutoff points identifying overweight, or obesity were different for women (> 35%), and for men (> 22%)^29,30^. BAI cutoff points were also used to identify normal weight (21–35% for women, 8–22% for men), overweight (> 35–39% for women, and > 22–26% for men), or obesity (> 39% for women and > 26% for men)^4,29,30^.

**Figure 1:**
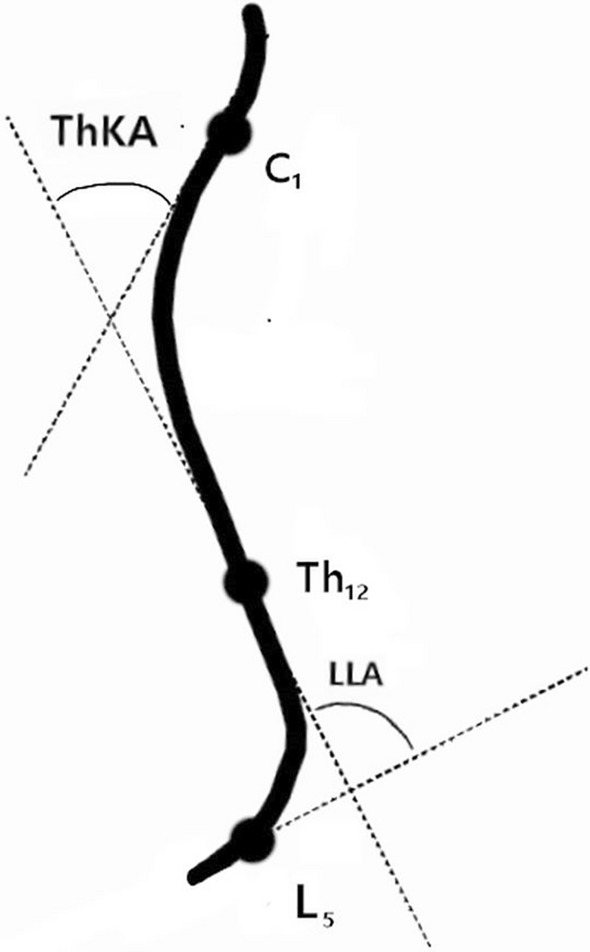
Diagram of thoracic kyphosis angle (ThKA) and lumbar lordosis angle (LLA).

Thoracic kyphosis angle (ThKA) and lumbar lordosis angle (LLA) were assessed using a Plurimeter-V gravity inclinometer (Dr. Rippstein, Zurich, Switzerland). The plurimeter is a precision-built, liquid pendulum inclinometer with a dial that can be fully rotated 360 degrees^5^. Based on Stolinski et al. study, this noninvasive technique was indicated as highly reliable and the correlation between sagittal spinal curvatures measured using a Rippstein plurimeter and digital photography was r = 0.949, p < 0.0001 for ThKA and r = 0.951, p < 0.0001 for LLA^31^. The participant was instructed to stand in a habitual position, with arms relaxed at the side of the torso, and feet shoulder-width apart. Measurement of ThKA was started by placing the zeroed plurimeter on the seventh cervical vertebra (C_7_) and the upper thoracic spine, and then the measurement was taken at the level of the thoracolumbar junction, above the apex of lumbar lordosis. The LLA was measured by placing the zeroed plurimeter on the sacrum below the apex of lumbar lordosis and then the measurement was taken at the thoracolumbar transition above the apex^5,32,33^. The measurements were carried out with an accuracy of 1 degree, and values of 25–35 degrees were assumed as normal for both angles^4,5,32^. We defined hyperkyphosis as a ThKA exceeding 35 degrees, and hypokyphosis as a ThKA below 25 degrees. Analogous values were adopted for the LLA, classifying the LLA above 35 degrees as hyperlordosis, and below 25 degrees as hypolordosis. A diagram of the ThKA and LLA is shown in Fig. 1.

A short Polish version of the International Physical Activity Questionnaire (IPAQ-SF) was used to assess total PA, time spent on vigorous-intensity PA (VPA), moderate-intensity PA (MPA), and walking as low-intensity PA (LPA)^34^. The IPAQ provides information about the frequency and duration of PA undertaken by the participants during the previous seven days (at work, at home, during leisure time, during active locomotion, etc.) at three levels of intensity specified in MET (metabolic equivalent of task), i.e. vigorous (8 MET), moderate (4 MET), and light intensity (3.3 MET), and sedentary time^34–36^. These metabolic equivalent of task (MET) values were used to calculate the weekly total physical activity (TPA) by considering the intensity and duration of different physical activities performed by an individual.

All participants were familiar with the IPAQ-SF and were asked to estimate the number of minutes spent in the previous seven days on VPA (described as > 6.0 MET), MPA (described as 3.0–6.0 MET), and walking (described as 3.3. MET)^35,37^. Only activities lasting at least 10 min (without a break) were recorded in the questionnaire.

The IPAQ has acceptable validity when assessing a level of PA in healthy adults^37^. The test–retest reliability of IPAQ was excellent. However, compared to accelerometers, concurrent validity for vigorous and moderate activities, walking, and sitting was low to moderate^38^.

Data collected from IPAQ-SF allowed the identification of participants who fulfilled the recommendations of the World Health Organization (WHO) regarding aerobic activity. As recommended, adults aged 18–65 years should do at least 150–300 min a week of aerobic MPA, or 75–150 min a week of aerobic VPA, or do an equivalent combination of aerobic VPA and MPA throughout a week^39^.

### Statistical analysis

The results are expressed as means with standard deviations (M ± SD), confidence intervals (− 95% to 95%), and minimal and maximal values. The normality of distributions and the homogeneity of variance were tested using the Shapiro–Wilk test and Levene’s test, respectively. Between-group differences in the quantitative variables were analyzed using the Mann–Whitney *U* test or *t*-test, as appropriate. Pearson’s chi-squared test was used for the analysis of qualitative variables. The effect size was evaluated using Cramer’s V (Pearson’s chi-squared test) as small effect (0.1 to < 0.2), moderate effect (0.2 to < 0.4), relatively strong effect (0.4 to < 0.6), strong effect (0.6 to < 0.8), or very strong effect (0.8 to 1); using Cohen’s d (*dc*) (*t*-test) as small effect (0.2 to < 0.5), moderate effect (0.5 to < 0.8), or strong effect (0.8 ≤); or using r index (the Mann–Whitney *U* test) as a small effect 0.1 to < 0.3, a medium effect 0.3 to < 0.5, and a large effect ≥ 0.5.

Associations between sagittal spinal curvatures, somatic parameters, and PA were assessed using Spearman’s or Pearson’s correlation coefficients. The Pearson correlation coefficient (*r*) and the Spearman’s rank correlation (rho) were qualitatively evaluated as follows: below 0.2–very weak correlation, 0.2 to < 0.4 as weak correlation, 0.4 to < 0.6 as moderate, 0.6 to < 0.8 as strong, and 0.8 to 1.0 as very strong correlation.

One-way ANOVA or Kruskal–Wallis ANOVA was conducted to investigate the association between not meeting/ meeting WHO recommendations (75–150 min VPA, and/or 150–300 min MPA)/meeting more than recommended (> 150 min VPA and/or > 300 min MPA) and sagittal spinal curvatures. The test was conducted separately for recommendation regarding VPA and separately for MPA. Additionally, one-way ANOVA or Kruskal–Wallis ANOVA was performed to investigate the association between the occurrence of hypokyphosis, hypolordosis, normal values of ThKA and LLA, hyperkyphosis and hyperlordosis in relation to PA.

The level of significance in all tests was set at α = 0.05. The statistical analysis was performed using STATISTICA ver. 13.3 Tibco Software Inc.

### Ethical approval and consent to participate

The study was approved by the Bioethics Committee of the Jerzy Kukuczka Academy of Physical Education in Katowice (No. 3/2012) and conformed to the standards established by the Declaration of Helsinki. All participants were informed about the type and aim of the study, and they provided informed consent before participating in the study.

## Results

### A comparative study of male and female students across studied parameters

**Figure 2:**
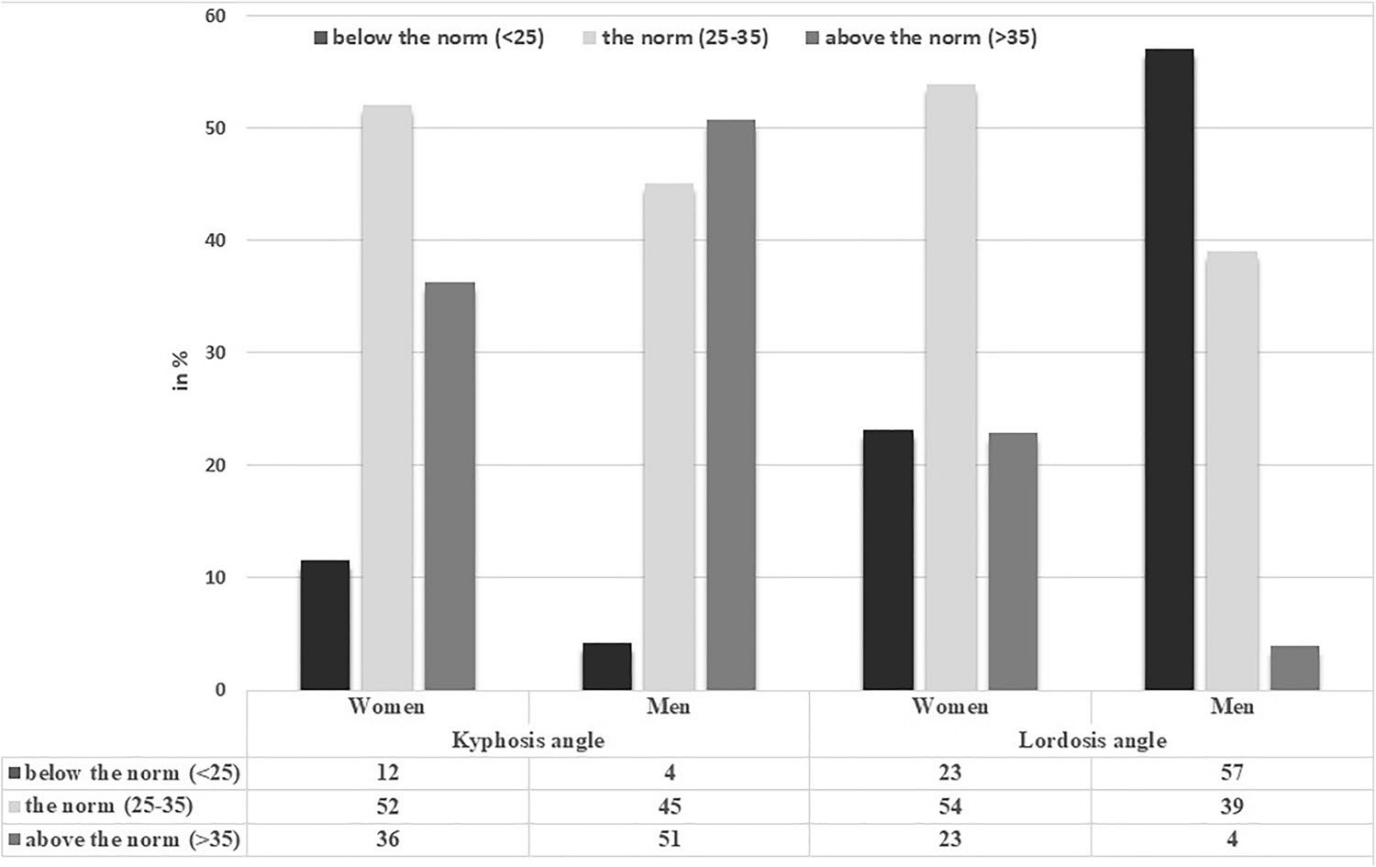
The occurrence of normal values of anteroposterior spinal curvatures, hypokyphosis, hyperkyphosis, hypolordosis, and hyperlordosis in students.

**Figure 3:**
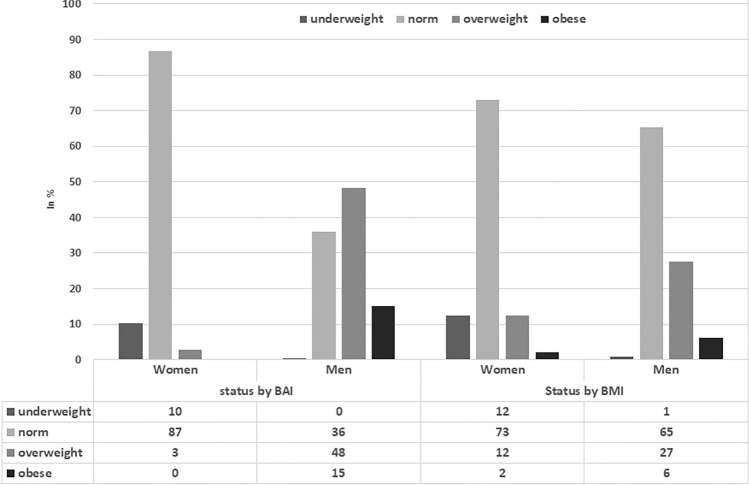
The occurrence of underweight, normal body mass, overweight, and obesity with respect to body adiposity index (BAI) and body mass index (BMI) in students.

Table 1 presents somatic parameters and sagittal spinal curvatures of the participants. Thoracic kyphosis angle (ThKA) was significantly lower in women than in men (p < 0.0001, r = 0.22), whereas lumbar lordosis angle (LLA) was significantly higher in women than in men (p < 0.0001, r = 0.42) (Table 1). The occurrence of normal values of thoracic kyphosis, hypokyphosis, and hyperkyphosis differed depending on gender (*Chi*^2^ = 16.25, p = 0.0003, V = 0.17). The occurrence of normal values of lumbar lordosis, hypolordosis, and hyperlordosis also differed between women and men (*Chi*^2^ = 80.61, p < 0.0001, V = 0.37) (Fig. 2). The analysis showed statistically significant differences in all somatic parameters (p < 0.0001) between women and men except hip circumference (Table 1). There were also statistically significant differences in the occurrence of underweight, normal body mass, overweight, and obesity depending on BAI (*Chi*^2^ = 274.4, p < 0.0001, V = 0.68) and BMI (*Chi*^2^ = 46.2, p < 0.0001, V = 0.28) between male and female students (Fig. 3).

**Table 1:** Somatic parameters, thoracic kyphosis (ThKA) and lumbar lordosis (LLA) angles in female and male students.

Somatic parameters and spinal curvatures	Female students (n = 380)			Male students (n = 211)		
	M ± sd	C.I. – 95–95%	Range	M ± sd	C.I. − 95–95%	Range
BH [cm]	166.24 ± 6.22**	165.62–166.87	150–185	180.54 ± 6.64**	179.64–181.45	160–198
BM [kg]	60.24 ± 9.96**	59.24–61.25	39–111	78.69 ± 12.87**	76.94–80.44	53–137
BMI	21.76 ± 3.10**	21.44–22.07	15–38	24.11 ± 3.49**	23.63–24.58	17–36
Fat [%]	25.61 ± 7.10**	24.89–26.33	4–49	14.95 ± 6.16**	14.11–15.78	3–38
WC [cm]	71.92 ± 7.84**	71.13–72.71	58–112	83.46 ± 9.12**	82.22–84.70	67–118
HC [cm]	93.18 ± 7.83	92.39–93.97	68–125	94.25 ± 9.40	92.98–95.53	62–124
BAI	25.52 ± 3.65**	25.15–25.88	14–37	20.91 ± 4.03**	20.36–21.45	7–35
ThKA [^0^]	32.90 ± 7.26**	32.17–33.64	12–58	36.32 ± 7.66**	35.28–37.36	10–60
LLA [^0^]	30.14 ± 7.27**	29.40–30.87	5–52	23.64 ± 6.45**	22.76–24.51	8–50

*BH* body height, *BM* body mass, *BMI* body mass index, *WC* waists circumference, *HC* hip circumference, *BAI* body adiposity index, *ThKA* thoracic kyphosis angle, *LLA* lumbar lordosis angle.

**Statistically significant difference between male and female students at p < 0.0001.

Table 2 presents the PA of the participants at various levels of intensity (low, moderate and vigorous) in minutes [min], as well as the total PA in Metabolic Equivalent Task minutes [METmin]. Female students self-reported significantly lower VPA (p < 0.0001, r = 0.35), MPA (p = 0.004, r = 0.12), and TPA (p < 0.0001, r = 0.19) than male students (Table 2), whereas there was no significant difference in LPA.

**Table 2:** Self-reported physical activity of studied female and male students.

Physical activity	Female students (n = 380)			Male students (n = 211)		
	M ± sd	C.I. − 95%–95%	Min–max	M ± sd	C.I. − 95%–95%	Min–max
VPA [min/week]	139.51 ± 154.26**	123.95–155.07	0–1050	293.34 ± 230.33**	262.08–324.60	0–1200
MPA [min/week]	167.12 ± 229.71*	143.95–190.29	0–2520	190.44 ± 192.90*	164.26–216.62	0–1440
LPA [min/week]	639.11 ± 701.50	568.35–709.86	0–4200	556.78 ± 656.23	467.72–645.84	0–4200
TPA [METmin/week]	3893.61 ± 2826.00**	3608.56–4178.65	0–17,090	4945.86 ± 3097.17**	4525.54–5366.18	495–18,660

*VPA* vigorous-intensity physical activity, *MPA* moderate-intensity physical activity, *LPA* low-intensity physical activity, *TPA* total physical activity.

*Statistically significant difference between male and female students at p < 0.01, **Statistically significant difference between male and female students at p < 0.0001.

With respect to the WHO recommendations, nearly half of the female (37%) and male (46%) students self-reported their participation in MPA for at least 150 min per week, while 59% of women and 82% of men self-reported their participation in VPA for at least 75 min per week, with this difference being statistically significant (*Chi*^2^ = 48.5, p < 0.0001, V = 0.29).

Given the observed disparities in anteroposterior spinal curvatures, somatic parameters, and PA between women and men, we conducted further analyses stratified by sex.

### Specific outcomes for female students

The Kruskal–Wallis ANOVA test revealed a significant association between ThKA and LPA [min/day] (Kruskal–Wallis H = 6.35, p = 0.0418). Specifically, students with ThKA within normal values reported a higher amount of LPA per day (117 min), compared to those students with ThKA below (100 min of LPA per day) or above the norm (99 min of LPA per day). However, based on the Kruskal–Wallis ANOVA test, no statistically significant associations were found between the occurrence of hypolordosis, normal values of LLA, or hyperlordosis and PA. Additionally, no significant differences were observed between not meeting/meeting WHO recommendations (75–150 min VPA, and/or 150–300 min MPA)/meeting more than recommended (> 150 min VPA and/or > 300 min MPA) and sagittal spinal curvatures (Table 3). Furthermore, the correlation analysis between PA and sagittal spinal curvatures did not reveal any significant relationships.

**Table 3:** Sagittal curvatures of the spine in the context of meeting the WHO recommendations.

The WHO recommendations/groups		n	ThKA (mean ± sd)	Sig	LLA (mean ± sd)	Sig
Women (n = 380)						
VPA	Not meet	155	32.86 ± 7.47	0.6977	30.88 ± 7.38	0.1826
	Meet (75–150 min/week)	90	33.63 ± 7.55		29.53 ± 7.78	
	Meet (> 150 min/week)	135	32.47 ± 6.81		29.67 ± 6.76	
MPA	Not meet	239	32.85 ± 7.34	0.8467	30.03 ± 7.30	0.2235
	Meet (150–300 min/week)	85	32.66 ± 7.42		29.62 ± 7.27	
	Meet (> 300 min/week)	56	33.49 ± 6.70		31.38 ± 7.15	
Men (n = 211)						
VPA	Not meet	39	36.26 ± 7.01	0.0686	25.15 ± 7.01	0.5591
	Meet (75–150 min/week)	35	34.06 ± 8.35		23.20 ± 5.70	
	Meet (> 150 min/week)	137	36.92 ± 7.60		23.31 ± 6.45	
MPA	Not meet	113	36.70 ± 7.16	0.5344	23.88 ± 6.63	0.9361
	Meet (150–300 min/week)	59	36.56 ± 8.04		23.53 ± 6.07	
	Meet (> 300 min/week)	39	34.87 ± 8.47		23.08 ± 6.60	

*VPA* vigorous-intensity physical activity, *MPA* moderate-intensity physical activity, *ThKA* thoracic kyphosis angle, *LLA* lumbar lordosis angle, *sig*. significance in the Kruskal–Wallis ANOVA test.

Regarding the Kruskal–Wallis ANOVA test, significant associations were found between the occurrence of hypokyphosis, normal value of ThKA, hyperkyphosis and waist circumference (Kruskal–Wallis H = 12.08, p = 0.0024), BAI (Kruskal–Wallis H = 8.36, p = 0.0153), and fat percentage (Kruskal–Wallis H = 7.85, p = 0.0198). Additionally, significant associations were observed between the occurrence of hypolordosis, normal value of LLA, hyperlordosis and waist circumference (Kruskal–Wallis H = 11.23, p = 0.0036) and fat percentage (Kruskal–Wallis H = 7.55, p = 0.0229). Furthermore, the correlation analysis indicated that ThKA was positively correlated with BMI (rho = 0.14, p = 0.0074), BAI (rho = 0.14, p = 0.005), waist circumference (rho = 0.16, p = 0.002), and fat percentage (rho = 0.14, p = 0.0064), whereas LLA was positively correlated with BMI (rho = 0.13, p = 0.0116), BAI (rho = 0.11, p = 0.0278), waist circumference (rho = 0.17, p = 0.0008), and fat percentage (rho = 0.14, p = 0.0055). However, the observed correlations were very weak.

### Specific outcomes for male students

The Kruskal–Wallis ANOVA test did not reveal any statistically significant associations between the occurrence of hypokyphosis, hypolordosis, normal values of ThKA and LLA, hyperkyphosis, and hyperlordosis in relation to PA. Additionally, there were no significant differences observed between individuals who did not meet or met WHO recommendations for PA (75–150 min VPA, and/or 150–300 min MPA) and those who exceeded the recommendations (> 150 min VPA and/or > 300 min MPA) concerning sagittal spinal curvatures (as shown in Table 3). Furthermore, no significant associations were found between the occurrence of hypokyphosis, hypolordosis, normal values of ThKA and LLA, hyperkyphosis and hyperlordosis in relation to somatic parameters. However, the correlation analysis revealed a positive correlation between LLA and BMI (rho = 0.14, p = 0.0478) as well as waist circumference (rho = 0.17, p = 0.0163). In should be noted that observed correlations are very weak and should be interpreted cautiously.

## Discussion

This study assessed sagittal curvatures of the spine. Female students were characterized by less pronounced thoracic kyphosis and deeper lumbar lordosis than male students, and these differences were statistically significant with a small or moderate effect size, respectively. The occurrence of normal thoracic kyphosis, hypokyphosis, and hyperkyphosis differed significantly between women and men with a moderate effect size. The occurrence of normal lumbar lordosis, hypolordosis, and hyperlordosis was also significantly different with a relatively strong effect size. These findings are supported by previous studies. Kargarfard et al. revealed that both kyphosis and lordosis differed significantly between gender groups, with male students exhibiting larger ThKA and smaller LLA than female students^21^. Similarly, Poussa et al., Lang-Tapia et al. and González-Gálvez et al. also found that adolescent and adult male participants were characterized by a smaller lumbar lordosis and larger thoracic kyphosis than female participants^2,40,41^. In contrast, Tuz et al. based on their study involving 2154 male and female students, revealed statistically significantly smaller lumbar lordosis in men compared to women, while the authors found no significant differences in thoracic kyphosis^27^. Grabara observed that male students had significantly smaller lumbar lordosis than female students, whereas thoracic kyphosis did not differ between women and men^5^. Similar findings were obtained by Mirbagheri et al. among students^42^, and by Nourbakhsh et al. among women and men with and without low back pain between ages 20 to 65 years^43^. Janssen et al. in their study assessing spino-pelvic alignment in young adults, found no significant differences in thoracic kyphosis and lumbar lordosis between women and men. However, the authors observed a greater dorsal inclination of the thoracic and thoracolumbar spine in women compared to men^44^. The increased lumbar lordosis angles observed in women in our study and in studies by others authors may stem from variations in vertebral shape. Anatomical disparities and functional capacity also impact biomechanical factors during upright posture^2,43^.

The assessment of PA in this study revealed that female students were less likely to report VPA than male students, with a moderate effect size. Additionally, female students were also less likely to report MPA and TPA compared to male students, with small effect size. The gender disparity in participation in PA has been consistently observed in previous studies^45–49^. Specifically, Grabara et al. found significantly higher levels of VPA, MPA and TPA in male teachers compared to female teachers^47^. In a prospective study involving 412,413 U.S. adults, Ji et al. reported significantly greater engagement in MPA, VPA and muscle strengthening sessions per week among men compared to women^50^.

The present study investigated potential associations between spinal curvatures, self-declared PA, and somatic parameters. The study revealed that among female students, the ThKA was significantly associated with LPA. Women with ThKA values within the normal range reported higher levels of LPA compared to those with ThKA below or above the norm. However, no significant associations were found between spinal curvatures and MPA, VPA, TPA, or adherence to WHO recommendations. This lack of association was consistent across both female and male cohorts. Therefore, our hypothesis regarding the existence of a relationship between spinal posture and physical activity was not confirmed. Nevertheless, it is essential to highlight the benefits of regular exercise in strengthening the muscles that support the spine, thereby improving posture and reducing the risk of postural abnormalities. Targeted exercises, specifically those focusing on the core muscles (such as abdominals and back extensors), contribute to maintaining the spine’s natural curves^51^. The absence of associations between spinal curvatures and PA levels may suggest that the study cohort exhibited relative homogeneity in terms of both, PA levels and anterior–posterior spinal curvatures. This study did not include athletes but only students who engaged in PA on an amateur basis and not all participants adhered to the WHO recommendations for PA. Additionally, it is important to acknowledge that the PA assessment conducted using the IPAQ did not offer insights into the specific types of PA undertaken by participants, but rather provided information solely on the intensity and weekly volume of PA performed.

The results of previous studies are inconclusive regarding the relationship between the shape of spinal curvatures and PA. It usually depends on the population studied. Youdas et al. observed that lumbar lordosis was associated with PA in men older than 40 years without a history of back pain, but the authors did not find this association in women^52^. The opposite findings were reported by Nourbakhsh et al. who concluded that lumbar lordosis was not affected by the level of PA in subjects between ages 20 and 65 years^43^. Based on studies among athlete populations, it has been assumed that professional sports training may have both positive and negative effects on spinal curvature. Athletes in some sports have a deepened lumbar lordosis compared to the general population because of repetitive performance of movements (e.g. running) that causes a cumulatively increasing lumbar lordosis angle^16^. Other sports are characterized by a flexed posture of the trunk while sitting or standing, which can lead to hyperkyphosis, such as in adolescent canoeists^53^, adult male cyclists^54^, skiers^55^, and adolescent male volleyball players^56^. Rock climbers were characterized by increased thoracic kyphosis and lumbar lordosis^57^, whereas male and female dancers demonstrated significantly smaller thoracic kyphosis and lumbar lordosis angles than track and field athletes^20^. Hatha yoga practitioners were more likely to be characterized by normal or smaller thoracic kyphosis and lumbar lordosis than non-athletes, and yoga training was found to have a beneficial effect on posture^5^. Pilates exercises were also found a beneficial and appropriate form of PA to maintain sagittal spinal curvatures within normal values^18^.

The study revealed associations between spinal curvatures and somatic parameters. Specifically, it was found that female students with greater waist circumference, higher percentage of body fat and elevated BAI were more likely to exhibit deepened thoracic kyphosis. Additionally, female students with higher values of waist circumference and body fat percentage were more likely to have increased lumbar lordosis compared to those students with lower values of these variables. Furthermore, positive weak correlations were observed among women between ThKA and LLA with waist circumference, body fat percentage, BMI and BAI. Similarly, among men, there were weak correlations between LLA and waist circumference as well as BMI. Therefore, it can be inferred that certain somatic parameters may contribute to the deepening of anterior–posterior spinal curvatures, partially confirming our hypothesis. These findings underscore the importance of considering somatic factors when assessing spinal posture in students.

Previous studies have yielded inconsistent results concerning the associations between thoracic kyphosis, lumbar lordosis, and various somatic parameters. Kargarfard et al. and Hoseinifar et al. in their study conducted among students, did not identify significant relationships between thoracic kyphosis and BMI within gender groups^21,24^. Interestingly, yoga practitioners exhibited positive correlations between thoracic kyphosis and body mass, BMI, as well as age^5^. In a study involving 742 female students and 539 male students, Zwierzchowska et al. observed statistically significant differences in LLA, but not in ThKA in both groups of women and men classified according to BAI cut-off points. Specifically, as LLA increased, BAI also increased^4^. These findings align with our own results related to women. Zwierzchowska et al. reported no significant differences in ThKA and LLA in women and men classified according to BMI cut-off points^4^. Similarly, Romero-Vargas et al. did not observe differences in LLA when comparing BMI-based groups^23^. This suggests that BMI alone may not be a reliable predictor of spinal curvature. Contrary to the above findings, our study revealed a weak correlation between LLA and BMI in both women and men. Consistent with our observations, Hoseinifar et al., Kargafard et al. and Onyemaechi et al. reported a significant, positive relationship between lumbar lordosis and BMI within gender-specific groups^21,24^ and for both sexes^25^.

### Strength and limitations of the study

The study was conducted on a relatively homogeneous group of young adults, who were of similar age, in a stable period of posturogenesis, and were full-time students at the same university. Additionally, they attended similar PA classes offered by the university. This homogeneity of the sample may enable a more precise and accurate analysis of the relationships between the variables of interest. Furthermore, since they were full-time students, none of them were employed full-time, thus eliminating the potential influence of occupational work on the variables under investigation.

This study has several limitations that should be considered when interpreting the results. The primary limitation of this study is that the study's sample comprised only students from a single Polish university, which may limit the generalizability of the findings to broader populations. Another limitation is the use of the IPAQ-SF instead of an objective method. While this is a widely used and popular method, it has been shown to overestimate PA levels compared to objective devices^58^. The reliance on self-reported PA data could introduce recall bias, as participants may inaccurately recall or misrepresent their activity levels, despite the questionnaire used in the study being previously familiar to them. Additionally, the study did not consider the type of PA undertaken (e.g. resistance training, stretching, or aerobic training), nor did take into account the WHO recommendations regarding muscle-strength activity. Another limitation is the omission of factors such as lifestyle habits and physical fitness, which could potentially influence the associations after investigation. Additionally, the study is limited by the absence of more advanced and reliable measuring devices, such as the Spinal Mouse or others, for assessing sagittal curves with greater reliability and validity.

Future studies should incorporate data on the types of PA. This may enable separate analyses of sagittal spinal curvatures based on the type of PA undertaken. Conducting longitudinal studies or intervention trials using objective methods for PA assessment could elucidate the potential effects of PA interventions on spinal curvatures over time.

## Conclusions

Female students exhibited less pronounced thoracic kyphosis and deeper lumbar lordosis, and they also reported lower VPA, MPA and TPA than male students.

The results suggest the existence of associations between anterior–posterior spinal curvatures and certain somatic parameters, particularly among women. Surprisingly, we did not observe significant associations between spinal posture and VPA and MPA. However, a weak association emerged with LPA among women. These findings underscore the multifaceted character of spinal alignment, necessitating additional research across diverse population groups.

Based on this study it is reasonable to assume that maintaining a healthy body composition may help to reduce the risk of developing spinal curvature abnormalities. Young people are encouraged to participate in regular VPA and/or MPA and to maintain a healthy body composition to promote healthy spinal curvatures. It is important to note that these recommendations are general and may not apply to everyone.

## Data Availability

Data is available upon reasonable request at m.grabara@awf.katowice.pl.
